# Publishing Identifiable Patient Photographs in the Digital Age: Focus Group Study of Patients, Doctors, and Medical Students

**DOI:** 10.2196/59970

**Published:** 2025-03-05

**Authors:** Marija Roguljić, Dina Šimunović, Ivan Buljan, Marija Franka Žuljević, Antonela Turić, Ana Marušić

**Affiliations:** 1 Department of Periodontology University of Split Split Croatia; 2 Private Dental Practice Split Croatia; 3 Department of Psychology Faculty of Humanities and Social Sciences in Split University of Split School of Medicine Split Croatia; 4 Department of Medical Humanities University of Split School of Medicine Split Croatia; 5 Dental Outpatient Clinic Split Vis Croatia; 6 Department of Research in Biomedicine and Health Center for Evidence-Based Medicine University of Split School of Medicine Split Croatia

**Keywords:** patient photographs, patient privacy, confidentiality, data protection, ethical publishing, informed consent, open access, scientific journals, focus group.

## Abstract

**Background:**

The publication of patient photographs in scientific journals continues to pose challenges regarding privacy and confidentiality, despite existing ethical guidelines. Recent studies indicate that key stakeholders—including health care professionals and patients—lack sufficient awareness of the ethical considerations surrounding patient photographs, particularly in the context of digital scientific publishing.

**Objective:**

This qualitative study aims to explore how different stakeholders—patients, medical students, and doctors—understand the challenges of patient privacy and confidentiality in scientific publications. Additionally, it sought to identify key areas for future research, particularly in the context of online, open-access articles.

**Methods:**

We conducted 4 online focus groups due to COVID-19 restrictions: 1 with patients, 2 with final-year medical students, and 1 with head and neck physicians and dentists who regularly handle patient photographs. Participants were invited via email, and those who accepted took part in discussions lasting approximately 1 hour. All interviews were recorded and transcribed for analysis. All 4 focus groups were asked the same set of questions, covering the following topics: (1) consent for publishing patient photographs, (2) information on guidelines and standards for consent to publish patient photographs, (3) the importance of informed consent for various purposes, (4) methods for deidentifying patient photographs, and (5) the use of patient photographs in online, open-access publishing.

**Results:**

Three key themes emerged from the focus group discussions: (1) no definitive resources or practical recommendations available, (2) online publishing of patient images makes them more open to misuse, and (3) anonymization techniques have limitations. All stakeholder groups expressed a lack of knowledge about online publishing in general and concerns about the fate of patient photographs in the digital environment after publication. They emphasized the need for increased awareness among all relevant stakeholders and more stringent procedures for obtaining informed patient consent before publishing photographs. While they recognized the usefulness of image anonymization techniques in protecting patient identity, they were also aware that current methods remain insufficient to ensure complete anonymity.

**Conclusions:**

This qualitative study highlights that publishing patient photographs in open-access scientific journals is an important, serious, and largely unexplored issue, with all stakeholders still uncertain about the best ways to protect patient privacy. Clinicians, publishers, and journal editors should not only implement best practices to ensure fully informed patient consent for publishing identifiable photographs but also develop technical and governance safeguards. Future quantitative studies are needed to identify the most effective ways to enhance stakeholders’ knowledge, policies, and procedures, ultimately guiding the development of practical recommendations for the ethical publication of patient photographs in scientific journals.

## Introduction

Patient photographs are an essential tool in medical education and are routinely used in daily communication between health professionals to illustrate various clinical problems, procedures, treatment outcomes, and follow-ups. They are ubiquitous on social media platforms, websites, and in scientific journals; however, it is not always clear whether patient privacy is adequately protected in these outlets. As patient photographs are part of confidential medical documentation, their use must adhere to standards consistent with best ethical practices.

According to best practices outlined by the International Committee for Medical Journal Editors (ICMJE), it is recommended to avoid identifying patients in photographs whenever possible and to prioritize patient privacy [1]. The challenge lies in defining the boundary between nonidentifiable and identifiable photographs, particularly in the case of facial images. Traditionally, photographs of body parts or radiographs have been considered nonidentifiable and, therefore, do not require written patient consent. However, when such images are tagged with patient information in an article, they can become identifiable [2]. Additionally, artificial intelligence (AI)–based computer programs have demonstrated the ability to identify individuals from various types of patient photographs previously considered nonidentifiable [3].

Studies suggest that patients generally have a high acceptance of the use of their photographs for various purposes [4-6]. However, these studies also highlight the risk of privacy invasion through the disclosure of an individual’s identity. Several studies have shown that patients prefer the use of nonidentifiable photographs [4,6,7]. Additionally, patients are more likely to approve the use of their photographs for follow-up and education rather than for publications, social media, websites, or television broadcasts [8-10].

Although existing ethical standards for protecting patient privacy in the use of photographs are governed by various professional guidelines and legal regulations [1,11-13], a standardized practice has yet to be established. In a recent scoping review, we identified a wide range of disclosure practices for identifiable patient photographs and a lack of awareness of these practices and associated risks among both patients and health professionals [14].

The ethical publication of patient images is particularly relevant in the era of open-access journals and digital publishing [15,16]. For example, some Creative Commons licenses allow anyone to access and reuse parts or entire published scientific articles for any purpose, including commercial use, provided the source and authors are acknowledged [17]. Our previous study found that patients have a high level of trust in their doctors, often believing that verbal consent is sufficient or that permission is not even necessary to publish photographs of their bodies [18]. However, it remains unclear whether both patients and doctors fully understand the potential consequences of publishing identifiable patient photographs in open-access publications.

We conducted a focus group study with patients, students, and doctors—key stakeholders in scientific publishing—to further explore their views on publishing patient photographs in scientific journals, particularly in the context of open-access publishing.

## Methods

### Study Design

This qualitative study was conducted and reported following the COREQ (Consolidated Criteria for Reporting Qualitative Research) checklist for qualitative research (available in Multimedia Appendix 1) [19]. We conducted 4 focus groups using semistructured interviews with different stakeholder groups.

### Participants and Recruitment Procedure

Participants in this study included patients, students, and doctors from the University of Split School of Medicine (USSM) and the University Hospital of Split. We used purposive sampling to ensure the representation of different stakeholders across various roles in the research and publishing process:

*Patients* from the Department of Dentistry, University Hospital of Split, who had experience with diagnostic or therapeutic procedures related to the head and neck area, including participation in research studies.

*Doctors* with expertise in head and neck conditions from the Departments of Neurology, Dermatology, Maxillofacial Surgery, and Otorhinolaryngology at the University Hospital of Split, Split, Croatia.

*Students* in the final years of medical school at the USSM. We conducted 2 focus groups with medical students—1 with students from Croatia enrolled in the Croatian-language medical program and another with international students attending the English-language medical program. This distinction was made to capture perspectives from students with experiences in different health care settings (Croatia vs other countries, primarily within the European Union).

We contacted potential participants via email, inviting them to take part in a focus group discussion on research and publication ethics. In total, 30 individuals were invited to participate, and the groups were formed using a purposive sampling method. Each focus group was designed to achieve a balance in age and gender.

The focus group with non-Croatian students was conducted in English, while the other groups were held in Croatian.

### Focus Group Setting and Data Collection

Focus groups were conducted and recorded at the USSM during April and May 2020. The USSM is located in Split, the second-largest city in Croatia, with a metropolitan area population of approximately 350,000.

The focus group interviews were conducted online by 2 lead researchers: 1 female (MR) and 1 male (IB). As a result of COVID-19 restrictions on in-person meetings, we held virtual focus groups using the Zoom platform (Zoom Video Communications, Inc.).

The focus group questions were prepared in advance, and all 4 groups were asked the same set of questions, as outlined in the study protocol (Multimedia Appendix 2). Participants were asked about (1) their opinion on study findings showing that patients trust their doctors to publish identifiable photographs responsibly [18]; (2) sources of information on guidance and standards for obtaining valid informed consent; (3) different purposes for using patient clinical photographs; (4) anonymization of photographs; (5) consequences of publishing identifiable photographs in scientific journals; and (6) open-access publishing and licensing in relation to patient privacy protection.

Each focus group session lasted approximately 1 hour. The discussions were recorded using an audio-recording device and transcribed into a document file by one of the researchers (DŠ). The transcripts were then coded, and the codes were entered into a spreadsheet (Microsoft Excel).

### Research Team and Data Analysis

Most participants were familiar with the research team before the study began. The interviewer (MR), who served as the lead investigator, is a certified dental medicine doctor and an Assistant Professor at the USSM. Data analysis involved coding the transcripts, categorizing the initial codes, and identifying themes and patterns. Participant identities were anonymized by 1 of the investigators (DŠ). Each participant was assigned a code based on their focus group and a consecutive number as follows: F1, patients; F2, doctors; F3, medical students in the Croatian-language program; and F4, medical students in the English-language program, with individual participants numbered from P1 to P28.

Two researchers (MR and DŠ) conducted the initial coding of themes based on participants’ comments. Preliminary themes were identified during the analysis based on the focus group questions and were iteratively refined using a constant comparative approach [20], allowing for both the refinement of existing themes and the identification of new ones. During the initial coding, 1 researcher (DŠ) defined a large number of codes (>20), which were then reviewed and refined by 2 researchers (MR and AM) to reach a final consensus. Each code was assigned to a single theme category. Data saturation was reached after 3 focus groups, as the analysis of the third group did not yield any new themes. After deriving the themes, summaries of the identified themes were sent to participants for validation. Participants’ statements were translated from Croatian into English by 1 author (DŠ), checked for accuracy by another (MR), and finally reviewed by a professional translator. A selection of participant quotations is presented to illustrate the theme categories.

### Ethical Considerations

Ethics approval was obtained from the Ethics Committee of the USSM (Class: 003-08/20-03/0005, Reg. No.: 2181-198-04-20-0048) under the project funded by the Croatian Research Foundation, Professionalism in Health: Decision Making in Practice and Research (principal investigator: AM).

Before the focus group interviews, all participants were provided with an information letter and an informed consent form. This documentation outlined the study’s purpose, funding, recruitment process, methodology, potential risks or adverse effects, beneficiaries of the research findings, communication of results, data collection, analysis, and protection of personal information. It also informed participants of their right to withdraw from the study at any time and their opportunity to review and, if relevant, comment on interview transcripts and quotations. All participants signed the consent form after reviewing the study information. No compensation was provided for participation.

At the beginning of each focus group, participants were reminded about the confidentiality of any information shared during the discussion and were asked to avoid using identifying characteristics when describing their experiences. They were also informed that only the lead investigator, who conducted all focus groups, would have access to identifying data and that all data would be anonymized for analysis and publication.

## Results

### Overview

Thirty individuals were initially contacted and agreed to participate; however, 2 later withdrew due to online connectivity issues, resulting in a final total of 28 participants in the focus groups. There was a good balance of gender, age, and education level among participants in each focus group (Table 1), except for a wider age range among the patient group.

Three main topics emerged from the focus group discussions: (1) no definitive resources or practical recommendations available, (2) online publishing of patient images makes them more open to misuse, and (3) anonymization techniques have limitations.

**Table 1:** Characteristics of the participants in focus groups on publishing identifiable patient photographs

Characteristics	Patients (n=8)	Doctors (n=8)	Students of medical studies in Croatian (n=7)	Students of medical studies in English (n=5)
Gender (female/male), n	4/3	3/5	4/3	2/3
Age (years), median (range)	40.5 (24-61)	37.5 (28-48)	24 (24-25)	25 (24-25)
Country of origin	Croatia	Croatia	Croatia	Croatia (n=1), Germany (n=3), and United States (n=1)
Place of recruitment	Department of Dental Medicine, University Hospital of Split	Departments of Dentistry (n=4), Maxillofacial Surgery (n=1), Neurology (n=1), Dermatology (n=1), and Otorhinolaryngology (n=1) at the University Hospital of Split	University of Split School of Medicine	University of Split School of Medicine
Education level	Master’s degree (n=7), PhD degree (n=1)	MD degree (n=2), MD/PhD degree (n=6)	Final (6th) year of the study	Final (6th) year of the study

### No Definitive Resources or Practical Recommendations Available

The first theme highlighted the lack of clear best-practice recommendations for obtaining informed consent for the use of patient photographs in online journals. Participants expressed that universal guidelines, applicable to all cases and types of patient photographs, would be ideal. However, they also acknowledged the complexity and variability of different types of patient photographs and the contexts in which they are used, noting that not all images pose the same level of risk to patient privacy.

When asked about the resources they would turn to for best practices, participants expressed considerable uncertainty and gave varied responses. Some felt there should be someone they could consult but were unsure who that would be, while others suggested referring to journal-specific guidelines or seeking advice from their local ethics committee:

There should be a body that answers such questions. I am not sure which one it is or if it even exists. But there should definitely be someone to turn to.

F3/P23, student

Maybe on the site of one of the journals in which I want to submit my paper to. I think most of them have some kind of criteria for publishing patients’ data, so I would start there.

F4/P24, student

I would definitely check with the journal, check with somebody who had more experience, maybe Google and see what are like general guidelines.

F4/P27, student

...first, there are some instructions from the journal, the ethical commission and the ethical protocol. That is second, and after that, if we had to go further, I don’t know, we didn’t have the opportunity to have some kind of education about it.

F2/P16, doctor

Other participants suggested consulting colleagues or someone within their institution who might have the necessary knowledge, but again, no definitive source was identified.

I would also check in with more senior authors, there is someone else on the paper I am working with, maybe my supervisor or anyone who maybe has more experience. They would probably point me to some guidelines but I would also contact someone that I can talk to.

F4/P26, student

Well, we can informally consult with the ethics committee of Clinical Hospital Centre Split, I guess they know, they don’t have to give us anything officially, but we can just ask them as colleagues.

F3/P21, student

I guess they should appoint someone to deal with it.

F3/P22, student

The uncertainty and variability in responses indicated a general lack of awareness about reliable resources for publishing patient photographs in online journals. Building on this, participants emphasized the need for a clear and comprehensive set of recommendations and best practices to address this issue:

Well, maybe there should be some universal form that needs to be filled out and then it’s the same for everyone, so that we know, that we can’t make a mistake.

F3/P17, student

I think we should have a uniform attitude for any purpose of the image.

F2/P11, doctor

...you never know in the beginning what study might end up in a textbook so I think there should be one form of consent in the beginning of the study and once this is signed it should be OK either to publish into only the study or into the textbook.

F4/P28, student

Regarding the idea of definitive or universal best-practice recommendations, participants acknowledged that while desirable, creating such guidelines would be challenging due to the variability in patient photographs and the specific body parts that need to be shown in scientific publications. They provided examples, emphasizing that not all body parts carry the same level of sensitivity when publicly disclosed, potentially placing patients in more vulnerable positions to varying degrees.

Because most people don’t even care if it’s a picture of a tooth, nobody can connect him to it so he doesn’t care, on the other hand, when you send him a picture of your face, he is not indifferent.

F3/P18, student

A photo of the face is one thing, and a photo of the genitals is another. Let’s say these are two different categories within which a face could be seen according to some criteria.

F1/P6, patient

The complexity of the issue was further highlighted by participants who suggested that decisions should be based on the potential audience size for the photograph.

wherever that image is shown to a larger number of people, the greater the need for protection and the more detailed the consent should be.

F1/P6, patient

I think it’s actually about the number of people who will be seeing the image.

F1/P4, patient

Overall, participants’ responses suggested a lack of certainty and knowledge on how to obtain patient consent for publicly displayed photographs. One participant explicitly voiced this concern, stating, “I think it is something all of us need to get educated more on” [F4/P26]. This lack of awareness was further compounded by the absence of a universal or easily accessible resource to guide them, as well as the inherent complexity of the issue. Additionally, some doctors in the study believed they were not obligated to seek patient consent for using photographs, arguing that, as health care professionals, they were the most qualified to make such decisions.

As long as photographs are used in good and positive purposes, there is no need to obtain the consent for education, showing students, publication in journal. Of course, it implies that it will not be any type of abuse. It is mandatory to protect dignity and identity as far as possible. [F2/P14, doctor]

### Online Publishing of Patient Images Makes Them More Open to Misuse

Participants viewed the online publication of patient images as a complex issue, where patient privacy and photographs could be vulnerable to unregulated or illegal use. A key concern was the lack of control over how and where these images might be used. Additionally, participants highlighted that patients often lack sufficient knowledge to make fully informed decisions about digital publishing, including how images can be reused or the implications of different copyright licenses. Some proposed solutions included clarifying who is responsible for preventing misuse and finding ways to empower patients in their decision-making. Finally, participants—especially students—emphasized the crucial role of the doctor-patient relationship in ensuring proper informed consent and maintaining ethical integrity.

Despite existing copyright regulations, including requirements to credit the original material (eg, even under the most open Creative Commons license, CC BY 4.0), participants felt that patients and their images remained vulnerable to misuse.

People copy them [patient images] and take them for themselves and put them in their presentations when they need them for lectures and seminars.

F3/P21, student

So it makes a little difference to me because it refers to my private life, my private medical case or problem. So if such a picture is misused, I think it's a little worse than the one on the profile picture, which someone can cut out and put on something else.

F1/P4, patient

most of us have social networks and understand that a profile picture can be used for any purpose. Same thing [with pictures in journals].

F1/P1, patient

This potential for misuse was seen as higher in open-access journals:

Patients are losing their privacy in any case so even if you still say OK, we want to preserve the rights, technically you still end up doing it. The authors are giving up a part of their rights. They are also losing a part of protection of the patient.

F4/P26, student

In my opinion, it should be limited how the images of these scientific papers can be used, whether they are open-access or not. I mean, the man didn’t sign to be on some advertisement one day, he signed to be in a scientific magazine, so in my opinion it should be arranged a little, what can be done with that open-access.

F3/P18, student

I think the main difference is whether the journal is published online or not, is it open-access or not. I think that is a huge issue...If this photo is something that someone can find on the internet easily or it is a physical copy of the journal. This really changes their issue.

F4/P26, student

Some participants held a contrasting opinion, stating that they saw no distinction between open-access and closed-access publications, as most articles could still be illegally accessed through pirate websites.

Theoretically, we can find every locked paper on Sci-Hub and take those pictures, so as XX said, you signed that it can be used anywhere.

F3/P18, student

Participants noted that most patients were unaware of the implications of having their images published in an online article, including the meaning and impact of Creative Commons licenses. By contrast, some participants believed that patients might not be concerned with the details of how their images are used.

I don't think many people think about this.

F4/P26, student

Patients are not aware of the availability of their images.

F2/P9, doctor

I think it doesn’t matter what kind of license it is. I think it means nothing to the patient.

F2/P11, doctor

Patients are generally happy when they realize they have ended up in a scientific journal because it means that their case was really special. And then they brag, I am in a book, I am there, they showed my image at the congress.

F2/P13, doctor

Building on the idea that patients may be unaware of potential risks, some participants expressed the view that the responsibility lies with the manuscript author, physician, or the individual obtaining informed consent for image use. Some potential solutions to the risks of image misuse that were suggested were “warn the patient” [F3/P18, student], “to explain as much as possible to people because not everyone has the same amount of knowledge about the Internet” [F1/P4, patient], “communication with the patient, check everything” [F4/P26, student], and “check what the licences are that the journal has and maybe choose those that have a more strict criteria on further usage on the photos” [F4/P24, student].

Others said that the responsibility lies with the party that is culpable for the unauthorized use of the image, and suggested “arrange that it is punishable by law if someone’s image is published on an advertisement without permission” [F3/P22, student] and “it should somehow be regulated by law” [F3/P21, student].

A third proposed solution was to enhance patient autonomy by giving them greater control over how their images are used. For example, a participant suggested allowing patients to specify where their images could or could not be shared: “I [could] literally mark where I want and where I don't want [the image to be shared]” [F1/P4, student]. Another offered that one could “allow a certain deadline for withdrawing the image if the patient changes his mind within a week” [F1/P4, student], and a third participant suggested that “In the informed consent, put in a special box for publication for commercial purposes, that it can be published in open-access” [F3/P19, student].

### Anonymization Techniques Have Limitations

Building on the previous theme of patient images in online journals being vulnerable to misuse, a related concern emerged regarding anonymization techniques for photographs where the face is fully or partially visible. Participants expressed mixed opinions on whether a patient’s face could or should be protected using methods such as a black bar over the eyes. While some viewed these techniques as somewhat helpful, the overall sentiment was that they offer only limited protection and may not fully safeguard patient identities.

Some participants, particularly those from the patient stakeholder group, agreed that a patient’s identity should be protected as much as possible. As a result, they viewed anonymization techniques as a positive tool with the potential to at least partially achieve this goal:

I would put, I would use that anonymization system. Then I would feel less as if I was exposing the person.

F3/P19, student

I would be more inclined to mask the eyes, but with that tape, not with those two black dots. So, some kind of wide black band, such an option, but I would not show the patient completely.

F2/P9, doctor

I agree that I would feel more comfortable if I had black stripe over my face and I actually think [that], even though is not complete anonymization of the person, I feel like it is more abstract.

F4/P27, student

[With the black bar over the eyes] I am still not anonymous but at least I wouldn't feel totally exposed like not 100-percent exposed but 80% and that 20% of non-exposure would give me some kind of comfort.

F4/P25, student

Building on the idea that anonymization is not highly effective in protecting patient identity, some participants viewed these techniques as potentially diminishing the clinical value of the image, thereby undermining the purpose of including the photograph in the first place:

I am the first one who doesn’t even want to look at the image when I see an image with those things on patient’s eyes in books. It’s like I can’t see anything.

F3/P18, student

It’s a much better display without that anonymization, which doesn’t really have a function. And the patient, if he has already agreed, I don’t think it matters at all what the topic is because he was informed about the topic of the paper and about everything that is in the picture that he decided that it was OK for him. I don’t think there is any reason to cover his eyes.

F3/P21, student

I would rather publish it without it because it is somehow simpler...and is a better presentation of the case.

F3/P23, student

Additionally, not all cases were perceived the same in terms of anonymization techniques. Some participants distinguished between different patients in photographs or the conditions displayed:

But again, it depends on what we want to show. If you’re trying to show, I don’t know, the result of forehead wrinkle correction, then you have to show the eyes as well so that it can be better displayed all together. If we do anything aesthetically, if he already agreed, I would show everything.

F3/P18, student

It is some degree of anonymisation, not complete but still I would not want my face to be, you know, especially in some disease or something. (...) It depends on the condition.

F4/P26, student

Overall, participants’ accounts indicated uncertainty and ambivalence regarding the use of anonymization techniques. Few concrete suggestions were identified; instead, we observed a plurality of personal opinions and preferences on the subject. However, one participant suggested a possible way to reconcile the tension between clinical utility and patient protection: “to reveal only the part of the face that is important for it” [F1/P2, patient].

## Discussion

### Principal Findings

In this study, 3 main topics emerged from the focus group discussions, highlighting the need to improve existing guidelines and enhance stakeholders’ knowledge regarding the use of patient photographs in digital academic journals. While all participants recognized the challenges of maintaining patient privacy and confidentiality when using patient photographs, they were not fully aware of the potential consequences of publishing such images online, particularly in open-access formats. They viewed anonymization techniques as useful for protecting identity but also acknowledged their limitations in ensuring complete anonymity.

To the best of our knowledge, this is the first study to explore the knowledge and opinions of relevant stakeholders through focus group interviews to better understand the emerging issue of accessible identifiable patient photographs in the digital scientific environment. Previous studies were mostly cross sectional and relied on questionnaires with preset answers, which limited deeper insights into the problem [14]. This methodological approach offers a new perspective on the publication process of patient photographs in scientific journals. Our previous cross-sectional study, which examined opinions on obtaining consent for publishing patient photographs in academic journals, found that patients were generally lenient toward consenting to the publication of their facial photographs [18]. However, in this study, after focus group discussions that provided deeper insights into digital publishing and open-access formats, patients expressed a more rigorous stance on informed consent for the publication of their photographs compared with our earlier findings. They demonstrated a heightened awareness of the potential consequences of publishing their photographs in digital scientific journals and emphasized the need for full disclosure before providing written consent. This shift in perspective may, at least in part, be attributed to the higher education levels of the patients in this study. Furthermore, despite recognizing the limitations of fully anonymizing facial photographs by concealing the eyes, patients still considered it a useful method for providing at least some level of protection. Similarly, a study by Oh et al [21], which examined dermatology patients’ views on the clinical and nonclinical use of their photographs, found that patients were particularly concerned about privacy protection in nonclinical contexts, such as scientific publication.

When comparing the different stakeholder groups among the participants, we found that students were more aware of potential issues and had greater knowledge about sharing and publishing information online than patients and doctors. This was not unexpected, given their younger age and greater familiarity with digital technology. Recent studies on e-professionalism among medical and dental students and trainees have shown that they frequently use social media to communicate, learn, and share experiences, often by sharing information and patient data [22-24], which could include articles in open-access journals containing patient photographs. Discrepancies between students’ self-reported behaviors and their actual practices in social media activities have been noted, highlighting the need for clearer guidelines on ethical behavior in the digital environment [22]. Similarly, students in our study reported a lack of practical knowledge on how to ethically publish identifiable patient photographs in scientific journals, indicating that the future generation of doctors is aware of this issue. They also emphasized the need for clear and practical ethical guidelines on digital publishing in medicine and expressed openness to learning how to effectively protect their patients’ privacy in the digital environment.

In contrast to students and patients, medical and dental doctors predominantly believed that patients were unlikely to find or understand academic publications or the publication process in medicine. They considered themselves better positioned to make decisions on behalf of patients regarding the publication of their photographs. However, the doctors themselves demonstrated limited knowledge of available resources on ethical best practices in digital publishing. Although doctors are regarded as key stakeholders in this process [25], some believed that obtaining patient consent for using clinical photographs was not mandatory, and they were uncertain about how to address issues related to the misuse of such images. This raises concerns that doctors may not be adequately equipped to inform patients about the publication of their photographs in online journals or to obtain valid informed consent. The importance of obtaining such consent varies across countries [26,27], suggesting that cultural differences may influence ethical requirements for publishing patient photographs. Previous studies indicate that existing ethical guidelines are not uniformly implemented, highlighting the need for revision and improvement [14-16]. Additionally, publication requirements in scientific journals may sometimes be stricter than local ethical policies, potentially complicating the publication process. Furthermore, online publishing is evolving more rapidly than practical ethical guidelines are being reviewed, updated, and implemented, creating an ongoing challenge that requires continuous scrutiny and engagement. Particularly relevant is the increasing use of AI and deep learning methods in handling patient photographs. Several studies have already demonstrated the successful application of AI in detecting dental caries from patient photographs [28]. At the same time, concerns have been raised about the risks AI poses to patient privacy, as its use on photographs constitutes a form of secondary data usage that may not be adequately covered by existing privacy and patient protection guidelines [29]. A key risk associated with AI tools in dental imaging is the potential for patient reidentification or the inclusion of unprotected patient data in training data sets without proper consent. However, not all implications of AI are negative. An AI tool has been developed to generate a “digital mask” to enhance patient anonymity [30], demonstrating AI’s potential to help address some of the privacy and consent challenges associated with patient photographs.

Patient involvement in decision-making regarding diagnostic and treatment procedures is becoming increasingly important [27]. Qualitative studies exploring patients’ perspectives on the use of medical images—both for understanding recommended clinical procedures and for nonclinical purposes—have shown that most patients prefer to be involved in such decisions [21,31-33]. Although these studies had different research focuses, their findings align with those of our study, in which patients emphasized the importance of being fully informed before consenting to the publication of their photographs. In our study, participants also expressed concerns about the potential misuse of patient photographs, even when published under most open Creative Commons licenses. This highlights the importance of providing comprehensive information about all possible consequences. When developing practical guidelines for protecting patients with identifiable photographs in online scientific publishing, it would be advisable to identify key scenarios that require separate, targeted guidelines—considering factors such as the body part exposed and the applicable anonymization techniques. Similar to their involvement in medical treatment and research, patients should also be included as key stakeholders in the development of new guidelines on the ethical publication of their photographs in online scientific journals [34].

A limitation of this study is that the patient focus group included participants with a university degree, whereas survey studies investigating opinions on identifying photographs primarily included participants with a high school education [14]. The educational level can influence individuals’ opinions and knowledge about obtaining consent for the publication of identifying photographs in scientific journals. However, our study also highlights that even highly educated individuals, as well as young people—who are generally expected to be more proficient in using online information sources—still have limited awareness and understanding of the consequences of open-access publishing of identifying photographs. We also did not ask participants for their opinions on publishing identifiable photographs of children, which is a significant ethical issue, particularly in cases of rare and complex clinical conditions where sharing valuable information is crucial [35]. Additionally, the focus group interviews were conducted online rather than in person. However, this did not impact the quality of the discussion, as we used reliable technology that ensured high-quality audio transcripts, and participants were already accustomed to this format due to the COVID-19 pandemic [36]. Finally, while the study was conducted at a single medical school in a country, its findings can likely be generalized to many countries with publicly funded health care systems and to most countries in Europe, particularly in Central, Eastern, and Southeastern Europe [37].

### Conclusions

All stakeholders in our study—patients, doctors, and students as future doctors—lacked sufficient knowledge about best practices for the ethical publication of patient photographs in scientific journals, particularly when published in open-access formats. They also expressed considerable uncertainty about how to navigate such situations in practice. Even doctors at the university hospital were unfamiliar with the implications of open-access publication under open-access licenses, which suggests that they may be unable to adequately inform patients during the consent process to obtain valid consent for research. Based on these findings, it is crucial to enhance the education of health care professionals on the evolving digital landscape of publishing, different models of patient-doctor relationships, and ethical practices for protecting patient-sensitive data such as photographs—not only at the graduate education level but also throughout their professional careers.

Health care professionals should be equipped to adequately protect patients by discussing potential privacy violations when their photographs are published in open-access journals. Institutional review boards and research ethics committees should ensure that researchers address this critical ethical issue at the protocol stage of their studies. Publishers and journal editors should not only implement best practices to ensure fully informed patient consent for publishing identifiable photographs but also develop technical and governance safeguards. The increasing use of AI in handling patient photographs warrants special attention, as it introduces new and potentially unforeseen risks to patient privacy. However, AI tools also have the potential to enhance privacy protections and should be further explored for their utility in this context. This study may serve as a foundation for future interventional studies and expert panel surveys aimed at finding an appropriate balance between protecting patient privacy and facilitating the dissemination of new medical knowledge.

## Appendix

Multimedia Appendix 1 COREQ (Consolidated Criteria for Reporting Qualitative Research) checklist.

Multimedia Appendix 2 The Interview guide and interview questions for qualitative, focus group study.

## Abbreviations

AI: artificial intelligence
COREQ: Consolidated Criteria for Reporting Qualitative Research
ICMJE: International Committee for Medical Journal Editors
USSM: University of Split School of Medicine

## Data Availability

To preserve participants’ privacy and confidentiality, only anonymized transcripts of the focus groups are available from the authors on reasonable request.
