# Supplementary material for: Publishing Identifiable Patient Photographs in the Digital Age: Focus Group Study of Patients, Doctors, and Medical Students
Source: J Med Internet Res. 2025 Mar 5;27:e59970. doi: 10.2196/59970 (PMC11923485; doi:10.2196/59970)
Supplement: Multimedia Appendix 2 [file jmir_v27i1e59970_app2.docx]

**Appendix 2.** The Interview guide and interview questions for qualitative, focus group study.

**Interview guide**

*First, I would like to thank you for accepting our invitation to participate in this interview. As it was mentioned in the invitation letter, this interview will be conducted as a part of the ProDeM project (Professionalism in Decision Making in science and practice).*

*The aim of the project is to understand how decisions about health care and research are made in practice.*

*In this interview we would like to hear your opinions on the issues of publishing patient identifiable data in academic journals that are accessible online. Further, we would like to hear your opinion regarding the responsiblities and potential risks of launching publications associated with patient identifiable data.*

*I would like to point out that there are no right or wrong answers so please feel comfortable to express your opinion. Your opinion is very valuable to us and will contribute to the further development and the goal of the project.*

*This focus group is confidential; hence everything said will be used, as mentioned in the invitation letter, only for the purposes of the ProDeM project.*

*During the focus group, my colleague and I will take notes and the conversation will be recorded. The recording is only to ensure we have all your answers. The transcription will be anonymous, and tapes will be destroyed after a certain period (end of the project).*

Background description

Previously we conducted the study about stakeholders’ opinions about informed consent for publication of patient clinical photographs with different level of identifiability in academic journals. We included patients, students of the final two years of medicine and dental medicine and dental and medicine doctors. They all were surveyed with the same questionnaire that consisted of the most common clinical images such as X-rays, photograph of the hand, part of the face, photograph of the face with eye area blurred or covered and photograph of the face without any deidentification technique. They were asked about the type of consent a patient would need to provide for each type of image. We also asked them whether the patient should read the manuscript before giving consent for publication of a clinical photograph. The results of our study showed that Croatian patients considered that doctors were not required to ask them for permission about publication of their photographs in academic journals. Dental students and medical and dental doctors had similar opinions on this issue – although stricter than those of the patients, they were below current ethics standards for publishing research.

(present the figure from the article)

Research questions

The focus group study will be guided by the following research questions:

1. How do you explain the findings of this study – that patients often consider that the doctors do not have to ask them for permission to use their photograph in a publication or that it is sufficient to provide oral consent?

Probes:

- What would you expect to answer on this question?
- Why?

2. Where would you look for information about guidance and standards for consent for publishing patients’ clinical images?

- Which type of guidance would you considered as the standard in practice?

3. Patient’s clinical image could be used for different purposes (medical record, diagnostic aids and treatment planning, communication with colleagues, teaching and lecturing, research, publication (print and/or electronic media)). Are they equally important in regard to consent?

Probes:

- How would you rank the importance, or seriousness of informed consent for those uses?

(here the participants will jointly rank the possible uses of clinical images)

- Would your opinion change if clinical images were identifiable (e.g., full face) vs non-identifiable (e.g., panoramic y-ray of the teeth)

4. Current publishing standards state that masking the patient’s eyes in a full face photograph is not anonymization and it not necessary. It is important to obtain patient’s consent for the publication of the image and then publish it without alteration. Do you agree with this practice? What would you prefer to be done with your own identifiable clinical image? Which type of anonymization would you consider as the most effective?

Probes:

- Would you feel more comfortable if your eyes were covered even if you can be easily identified?

5. What happens with patients’ clinical images that have been published in academic journals?

Probes:

- What patients’ clinical images can be used once they are published? Do you know of any examples?

6. When a clinical image is published in open access licence CC BY4, anyone can use it for any purpose. CC licences are a form of copyright licence, where a part of researcher’s copyright is given up for the public use. It is stated that this giving up of publishing rights does not override other rights. Does this include patient’s right to privacy?

Probes:

- When a patient gives consent for publishing an identifiable clinical image in a publication under CC BY4 licence, does he or she gives up his or her right for privacy?
- Are patients aware of what can happen with their clinical image once it is published?
- Whose responsibility it is to ensure that patients are fully informed about consent for publishing their photographs? What is the responsibility of the patient, the researcher, the journal/publisher, the reader?
